# A Case of the Luxation of the Femur Anteriourly and Upwards

**Published:** 1803-12-01

**Authors:** 


### *A* CASE *of the* LUXATION *of the* FEMUR ANTERIOURLY *and* UPWARDS. *Communicated by our Correspondent at Paris.*

THE following rare Case is inserted in the Recueil Periodique of the Medical Society of Paris; and Dessault, in his surgical works, give an account of a similar one, the success of which contradicts the unfavourable prognostic generally formed in these cases. It may be interesting to know some of the details, as well with respect to the appearances as to the mode of reduction employed.

A porter in a public office, aged 53, strong and robust, carrying a heavy burthen on his shoulders, slipped and fell with force on his right knee; but the direction of his fall was such as to drive the thigh backwards, while the body charged with the weight was carried in such a direction as to form a very acute angle with the femur, which was pressed backwards; at the moment of the fall the person complained of an extremely acute pain in the right groin, and was incapable of raising himself or moving the thigh. In

In this state he was carried to the Charity Hospital, where he was examined by the surgeon in attendance, who remarked the following appearances. The right inferior extremity was shorter than the other upwards of six lines, and much turned outwards, considerably more than in the fracture of the neck. Any motion in the part was impracticable; in the right groin a tumour was perceived on which the integuments rolled easily, which was found to be the head of the femur pushed from its cavity and resting on the pubis; the crural vessels lay withinside, but there appeared no swelling or tension of the part, and the circulation seemed to be carried on, though to a certain degree impeded; the patient complained loudly of pain in the groin; the buttock appeared flattened, and the great trochanter was carried forwards; in short, a dislocation of the bone anteriourly and upwards was ascertained, and the reduction was immediately attempted in the following manner. The patient was placed on a mattrass in a bed without posts, so that the assistants should have free liberty; a sheet folded on itself was placed on the inferiour part of the leg, above the malleoli, with a view to extension; the counter-extension was made by another placed in the opposite groin, having previously applied a cushion in order to moderate the pressure of the sheet during the extension; the two heads of this lac were directed towards the opposite side by passing over the loins and abdomen. A third lac was applied with a view to fix the pelvis on the crista ilei, and the two heads were brought to the other side, crossing the former, making with it an angle more or less acute, but the diagonal of which was compleatly in the direction of the luxated member. Four assistants made the extension, two the counter extension, and two fixed the pelvis; the surgeon was placed on one side, and while the assistants drew with all their might the respective lacs, the surgeon pressed with the palms of his hands the head of the bone downwards and backwards, at the same time endeavouring to bend the thigh on the pelvis. After two fruitless trials he suspected the insufficiency of the extensive force, and by adding two to the inferior, and one to the superior, the head of the bone was returned into its cavity with a very remarkable noise. All the parts immediately resumed their natural appearance, the motions though painful were effected with facility, the articulation was covered by a poultice, and in three or four days the patient left the hospital.

The case alluded to in Dessault's works, bears the closest analogy analogy in every circumstance to the one just related. The process of reduction however offered some varieties which it may not be unimportant to notice. Dessault conceived that the return of the bone was prevented by the small opening in the capsular ligament, and judged it necessary to lacerate and enlarge this opening by giving to the member different strong and painful motions; he made his extension from the ancle; and it may be useful here to remark, that this practice is universal among French surgeons, to make the extension from the extremity of the member in the reduction of all dislocations; and though contrary to the practice, as generally established in England, we believe the principles on which the French practice, or rather the old practice is founded, to be more conformable to the laws of mechanics,—but this by the way. Dessault also made his counter extension different from that we have just described, in as far as he carried his lac placed in the opposite groin across the patient's body, and over the opposite shoulder; the consequence was, that the body, dragged out of the line of direction of the extremity, the extending power lost so much of its force; a circumstance avoided by the application of two lacs acting in a diagonal line, and in the direction of the body or of the extremity. We may observe, that the bad consequences apprehended from compression of the vessels, do not in fact exist, though it is likely that the great pain proceeds from the pressure of the bone against the crural nerve. The weakness of the joint, said to exist through life, does not appear to be founded in fact.

